# The Impact of Morphological Awareness on Word Reading and Dictation in Chinese Early Adolescent Readers With and Without Dyslexia

**DOI:** 10.3389/fpsyg.2018.00511

**Published:** 2018-04-12

**Authors:** Sylvia Chanda Kalindi, Kevin Kien Hoa Chung

**Affiliations:** ^1^Faculty of Education, The Mount Saint Vincent University, Halifax, NS, Canada; ^2^Department of Early Childhood Education and Department of Special Education and Counselling, Centre for Child and Family Science, The Education University of Hong Kong, Tai Po, Hong Kong

**Keywords:** morphological awareness, cognitive-linguistic skills, word reading, word spelling, Chinese language, dyslexia, early adolescence

## Abstract

This study investigated the role of morphological awareness in understanding Chinese word reading and dictation among Chinese-speaking adolescent readers in Hong Kong as well as the cognitive-linguistic profile of early adolescent readers with dyslexia. Fifty-four readers with dyslexia in Grades 5 and 6 were compared with 54 chronological age-matched (CA) typical readers on the following measures of cognitive-linguistic and literacy skills: morphological awareness, phonological awareness, visual-orthographic knowledge, rapid naming, vocabulary knowledge, verbal short-term memory (STM), Chinese word reading, and dictation (or spelling). The results indicated that early adolescent readers with dyslexia performed less well than the typical readers on all cognitive-linguistic and literacy measures except the phonological measures. Both groups' scores showed substantial correlations between morphological awareness and Chinese word reading and dictation. Visual-orthographic knowledge and rapid naming were also associated with dictation in early adolescent readers with and without dyslexia, respectively. Moderated multiple regression analyses further revealed that morphological awareness and rapid naming explained unique variance in word reading and dictation for the readers with dyslexia and typical readers separately after controlling readers' age and group effect. These results highlight the potential importance of morphological awareness and rapid naming in Chinese word reading and writing in Chinese early adolescents' literacy development and impairment.

## Introduction

Developmental dyslexia, also referred to as reading disability, is a disorder characterized by severe and often pervasive difficulty in learning to read and spell despite normal intelligence and in the absence of sensory and neurological impairment or environmental deprivation (Lyon et al., 2003; Rose, 2009). Widely accepted as a life-span disorder, dyslexia has been well-studied in young children across languages (e.g., Snowling, 2000; McBride, 2015), although few studies document the progress of readers with dyslexia in adolescence (e.g., Wolf and Katzir-Cohen, 2001; Snowling et al., 2007), particularly for non-alphabetic languages. While several cognitive-linguistic skills are recognized to be important for reading development, it appears that the role of morphology in relation to other cognitive-linguistic skills could be of particular importance in enhancing the understanding of reading development and impairment in Chinese (e.g., McBride-Chang et al., 2003; Shu et al., 2006). Although not discounting the role of phonological awareness in Chinese literacy development (e.g., Ho and Bryant, 1997), it is worth highlighting why morphological awareness could be of particular importance for literacy development in Chinese. The present study seeks to extend this work by investigating the development of cognitive-linguistic skills associated with both reading and spelling abilities during the transition from childhood to early adolescence among Chinese students with dyslexia. We drew on previous work to look at six cognitive-linguistic skills associated with both reading and writing ability in dyslexia across alphabetic and non-alphabetic languages, namely, morphological awareness, rapid naming, visual orthographic knowledge, verbal short-term memory (STM), phonological awareness and vocabulary skills (e.g., Ho et al., 2002; de Jong and van der Leij, 2003; Shu et al., 2006). The goal of this study is to investigate the potential importance of morphological awareness in relation to word reading and dictation among Chinese-speaking students with and without dyslexia in Hong Kong senior primary schools. Also to be examined, are the cognitive-linguistic skills that might distinguish Chinese early adolescent readers with and without dyslexia in Grades 5 and 6, as well as the relations among the six cognitive-linguistic skills, word reading and dictation in readers with and without dyslexia.

Below, we highlight literature regarding the cognitive-linguistic deficits of readers with dyslexia across alphabetic languages and the features of Chinese language. Later, we zero-in on why morphological awareness in relation to other cognitive-linguistic skills could be of particular importance in enhancing word reading and dictation among Chinese adolescent readers.

### Dyslexia in alphabetic languages

Although reading impairments can be found in all languages, the differences in languages, including their structure, indicate that dyslexia manifests itself differently (e.g., McBride, 2015). Extensive studies on dyslexia in alphabetic languages such as English indicate that dyslexia is primarily due to phonological processing deficits that affect the processing of speech sounds in a word, and it disturbs the learning of letter knowledge, resulting in failure to acquire adequate word recognition skills (e.g., Snowling, 2000; Spafford and Grosser, 2005). Catts and Kamhi (2005) further noted that those with dyslexia often have difficulty acquiring letter-sound correspondences and using such knowledge to “decode” unfamiliar words in a text once they begin learning to read. This difficulty with phonological sensitivity highlights how a lack of understanding of the close link between script and sound may interfere with the children's ability to read text accurately, eventually impacting reading comprehension. The second core deficit in dyslexia is deficient speeded processing (Bowers and Wolf, 1993; McBride-Chang and Manis, 1996). Measured via rapid automatized naming, deficits in rapid naming may interfere with the automatic processing that encompasses orthographic pattern extraction in a language (e.g., Bowers and Wolf, 1993). The lack of fluency tends to be more prominent among students with dyslexia acquiring literacy skills in a transparent orthography such as Finnish, where readers have an intact phonological decoding ability but with slow reading speed. On the other hand, readers with dyslexia acquiring literacy skills with a deep orthography, such as English, could exhibit difficulties in both phonological decoding and speed of reading (Aro and Wimmer, 2003). Besides these two deficits, readers with dyslexia may also exhibit difficulties in visual-orthographic knowledge (Corcos and Willows, 1993), including complications with morphological awareness, i.e., the sensitivity to morphemes in words (Carlisle, 1995; Leong, 1999). These problems generally impact the learners' ability to manipulate the structure of words, encode printed words, and analyze the meaning of words that could be morphologically complex. In fact, literacy learning in senior primary grades (e.g., 4th–6th grade) tends to be much more difficult for children because of the need to read and comprehend complex text as well as literature that demands more-challenging comprehension and critical thinking skills than merely grasping the alphabetic principle to enhance the decoding of words and familiar text. Most countries consider 4th grade to be a vital transition point into senior primary school because of pedagogical changes such as the shift from focusing on word decoding to reading comprehension (e.g., Chall, 1983). While this phase could be problematic for all students, it is likely that those with dyslexia could have an even more devastating experience. This brief overview of dyslexia in alphabetic languages suggests that multiple cognitive-linguistic causes are at play in students with dyslexia. Yet despite the distinctive features of writing systems such as Chinese, relatively few studies have shown interest in ascertaining what underlies dyslexia in these non-alphabetic orthographies.

### Characteristics of the Chinese language

Here, we shall describe certain features of Chinese, focusing on how language- and orthography-related aspects of Chinese could be different from alphabetic languages such as English. Highlighting these features is cardinal in terms of providing a basis for the importance of morphological knowledge in Chinese readers, especially those with dyslexia.

First, Chinese has sometimes been regarded as a morpho-syllabic writing system, because in Chinese, each basic graphic unit, referred to as a character, is connected with a morpheme (meaning unit) and signifies a spoken Chinese syllable (Mattingly, 1984; DeFrancis, 1989). A character is composed of strokes that are combined to form stroke-patterns, radicals, which are then used to make whole characters. As Guo (1995) notes, a whole character can have as many as 32 individual strokes. A character typically consists of two different parts, a semantic radical and a phonetic radical (Shu and Anderson, 1997). The phonetic radical provides clues to the sound of a character, although this information is unreliable when compared to the phonetic cues found in alphabetic orthographies, where each graphic unit, the letter, is directly linked to the sound (McBride, 2015). Alphabetic languages such as English are considered relatively transparent with regard to mappings between phonology and orthography, making phonological awareness critical for reading acquisition in alphabetic orthographies (Wimmer and Goswami, 1994; Aro and Wimmer, 2003). For Chinese, however, the relatively arbitrary associations between sound and print indicate that the orthography is not phonologically reliable but further requires that numerous characters and phonological units be stored in the lexicon. Thus, phonological awareness may not be as important to promote word reading in this script (Ho et al., 2004; Chung et al., 2010).

As alluded to earlier, the semantic radical is another constituent of a Chinese character, which unlike the phonetic radical, provides more-dependable cues that directly connect with the meaning of a character (Chung and Ho, 2010). Considering that in 80% of Chinese characters, semantic radicals are directly connected to meaning (Shu et al., 2006), some researchers argue that the structure of a Chinese character by itself likely points to the significance of morphological skills in Chinese reading (e.g., Lee et al., 2006). However, as indicated by McBride (2016), this is still an issue of debate. In any case, the direct association between the semantic radical and a character's meaning distinguishes morphological from phonological information in ways that are specific only to Chinese.

In addition to the above-highlighted functional regularity, the positional regularity of radicals is at the center of a character's orthographic structure and plays an important orthographic processing role (Shu and Anderson, 1997). Radicals typically occupy certain positions within a character. For example, the semantic radical mostly occupies the top or left portion within the top-bottom or left-right structure (for details, refer to: Chung and Ho, 2010; Kalindi et al., 2018). Thus, sensitivity to orthographic structures and radicals is a crucial factor influencing reading acquisition and failure in Chinese (Ho et al., 2003; Chung et al., 2011). In fact, aspects of inconsistency in the positional regularity of radicals, as highlighted by Ho et al. (2003), imply a likely complication for typical learners' literacy acquisition process (e.g., Tong et al., 2009) and a further heightened difficulty for those with reading difficulty in achieving competent reading levels (e.g., Chung et al., 2011). It is worth contrasting here that while Chinese learners have to acquire numerous characters and be conversant with the positional regularity of the radicals in order to develop literacy skills, learners in alphabetic languages simply have to learn a limited number of alphabet letters (for details, see McBride, 2016) that are always presented in a linear fashion. This, however, also presents considerable difficulty for those with reading difficulties (e.g., Bowers and Wolf, 1993).

Besides the orthographic features highlighted above, Chinese has some structural language features that affect typical and dyslexic readers differently, further highlighting the importance of morphological knowledge. Lexical compounding is one structural feature of the Chinese language that actually highlights the importance of morphological awareness in literacy acquisition (Shu et al., 2006). Typically, word formation in Chinese occurs by way of compounding two or more morphemes, and thus, many words share the same morpheme and are semantically related. Based on the language structure, Chinese can be said to be relatively semantically transparent because complex vocabulary can be built by combining morphemes via compounds. Although some level of lexical compounding is evidently common in alphabetic languages, it is worth noting that there is considerable variability among the alphabetic languages. For example, while lexical compounding is very common in Finnish, with some words ending up with as many as 50 letters, the English language does not allow for such compounding (for details see: McBride, 2016).

Closely linked to the feature of lexical compounding in relation to morphological awareness and literacy development in Chinese is homophone knowledge (Liu et al., 2013). In Chinese, the majority of words are multi-syllabic, with a further two-thirds of the words being bisyllabic (Taylor and Taylor, 1995). Thus, Chinese has largely been recognized as have a large number of homophones, a feature that is key for word recognition. To read competently, it is important for one to be sensitized to the different homographic and homophonic morphemes and to find the means to determine a character by considering it in context. Although this feature is evidently present in alphabetic orthographies such as English (for details see, McBride, 2016), the presence of homographs and homophones in Chinese is much more pronounced, thus highlighting the need for such skills. As Carlisle (1995) notes, the more children read, the more they understand about how morphemes are related to one another (Carlisle, 1995), and this further facilitates vocabulary development. In Chinese, learning to orally distinguish the many homophones may be bidirectionally associated with character recognition in typical readers (McBride-Chang et al., 2003). Chinese readers with dyslexia generally tend to perform poorly in morphological awareness tasks (e.g., Chung et al., 2010).

Given these features of Chinese, examining the cognitive-linguistic profile of dyslexia in Chinese is essential, while bearing in mind the highlighted features of Chinese that make our first goal worth exploring.

### Cognitive-linguistic profile of dyslexia in Chinese

Compared to research on dyslexia in alphabetic languages, research on Chinese literacy development, including impairment, is still in its infancy, and there is generally a paucity of studies on dyslexia. Moreover, similar to the trend in alphabetic languages, very few studies have looked at dyslexia in senior primary grades. Nonetheless, existing studies indicate that Chinese children with dyslexia present various underlying multiple deficits in cognitive-linguistic skills such as rapid naming, morphological awareness, phonological awareness, visual-orthographic knowledge, vocabulary knowledge, and verbal short-term memory (STM) (e.g., Shu and Anderson, 1997; Ho and Lai, 1999; McBride-Chang et al., 2003; Ho et al., 2004; Shu et al., 2006; Chung et al., 2010). Although some recent studies have identified rapid naming, visual-orthographic skills and morphological awareness as the most dominant types of cognitive-linguistic deficits in Chinese children with dyslexia (Ho et al., 2002, 2004; Shu et al., 2006), we present below why these cognitive-linguistic deficits, including those that may not be so “dominant,” could be of particular importance among readers with dyslexia in senior primary grades. Senior primary school is when learners transition to early adolescence and become more accustomed to “reading to learn” as opposed to “learning to read” (for details, see Chall, 1983). As such, our focus on readers in the 5th and 6th grades could reveal some developmental differences in the associations between cognitive-linguistic skills and literacy performance.

Given the various aspects of morphological awareness that are of practical importance to Chinese literacy acquisition, we measured the morphological awareness skills of early adolescents in the present study. Research in alphabetic languages indicates that morphological awareness significantly contributes to reading processes, especially with regard to reading problems (e.g., Casalis and Louis-Alexandre, 2000; Lyytinen and Lyytinen, 2004). Although recent studies conducted in Mainland China (e.g., Shu et al., 2006) have indicated the importance of morphological awareness in distinguishing children with reading difficulties from those without any such impairments, we felt it is important to still determine whether this measure would distinguish between poor and good adolescent readers in a different Chinese society, such as Hong Kong. Although the study by Shu and colleagues also focused on pupils from senior primary schools, it was conducted in Mainland China, and owing to some literacy-related differences between Hong Kong and Mainland China, we were curious whether our study would reveal any novel findings. It is worth pointing out here that while Mainland China uses a modified Chinese script, Hong Kong uses the traditional script, which is likely influence the reading process (for details, see Kalindi et al., 2018) and may be a critical issue when learners are now expected to read in order to learn. Morphological awareness—conceptualized in Chinese as the capacity to distinguish meanings among morpheme homophones or the skill of manipulating and accessing morphemes in words with at least two morphemes—could still be key in distinguishing between poor and good readers in Hong Kong because of both the structural nature of the Chinese characters and the structure of the language itself, as earlier indicated. Note that while in early literacy studies of alphabetic languages, measures of morphological awareness substantially focus on the derivational and inflectional morphology (e.g., Deacon et al., 2007), in Chinese, measures of morphological awareness that have been identified as important for word recognition in early childhood (e.g., Tong et al., 2009) and early adolescence (e.g., Liu and Zhu, 2016) typically focus on lexical compounding and homophone/homonym awareness due to the nature of the Chinese script. Chung (2018) further illustrates that in Chinese, morpheme awareness is commonly measured via tasks of morpheme discrimination, morpheme construction and morpheme production. Considering the naturally enormous presence of lexical compounding and homophones in the Chinese language, it is likely that deficits in morphological awareness in relation to other cognitive-linguistic skills may be much more conspicuous. Although the very nature of Chinese words as having two or more characters, as well as the generally morphological nature of the Chinese language, could aid in performing literacy tasks such as dictation, constituent characters in Chinese words tend to actually be cued in a specific context (Tong et al., 2009). Therefore, insensitivity to the morphemic structures may well have devastating effects in the literacy acquisition of both young and adolescent readers (e.g., Chung et al., 2011). Besides the likely importance of morphological awareness in Chinese literacy acquisition being imbedded deeply, in an operational definition of Chinese, that is, “a meaning-centralized writing system” (Tong et al., 2009, p. 429), it is possible that the underlying connection between Chinese morphological awareness and literacy skills in Chinese could also be explained using the metalinguistic processing model (Bialystok and Ryan, 1985). In that model, the key component skills underlying metalinguistic and literacy development include knowledge analysis and control of cognitive linguistic processing. Since literacy development enhances the learner's ability to manipulate and analyze language (Chomsky, 1979), it is possible that this skill of accessing or manipulating language (e.g., morphemic structures) “fits” Carlisle's (1995) basic definition of morphological awareness and, as such, somewhat parallels Bialystok and Ryan's (1985) broader concept of analyzed knowledge and its associations with literacy skills. In any case, previous studies show that morphological production and discrimination tasks reliably predict Chinese word reading in both typical readers (e.g., McBride-Chang et al., 2003; Liu and Zhu, 2016) and readers with dyslexia (e.g., Shu et al., 2006; Chung et al., 2011), although in dictation tasks, less-consistent prediction in typical readers has been observed (e.g., Tong et al., 2009; Liu and Zhu, 2016).

In addition to morphological awareness, we included phonological awareness measures. Phonological awareness is important for reading acquisition across languages because of its focus on accessing and manipulating speech sounds. Measured at the syllable, onset-rime and phoneme unit level, phonological awareness has been demonstrated to be highly correlated with English and Chinese word reading (e.g., McBride-Chang and Kail, 2002). Phonological awareness in Chinese literacy development has revealed mixed results (e.g., Chow et al., 2005; Chung et al., 2010), partly due to the location of study participants and the type of script they need to learn, which influences the early literacy instruction method adopted by the teachers (for details, see Kalindi et al., 2018). In any case, we included measures of phonological awareness in this study because of previous demonstrations of the key role it plays in Chinese word reading and spelling among typical readers (e.g., Tong et al., 2009; Liu and Zhu, 2016) and those with dyslexia (Shu et al., 2006).

Another construct we considered important for distinguishing individual variability among Hong Kong children was rapid naming. Across orthographies, there is consensus on the significance of rapid naming as a core cognitive-linguistic skill for reading acquisition (e.g., Wagner et al., 1997; Ho and Lai, 1999). Profiling studies among Hong Kong children indicate the presence of multiple deficits in individual children, of which rapid naming and orthographic processing are the most dominant profile deficits, including visual and phonological processing (Ho et al., 2002, 2004). Considering that Chinese character recognition is a relatively “arbitrary” process (Manis et al., 1999), including a rapid naming measure may tap into the capability of learning arbitrary links between print and sound. Thus, based on previous profiling studies (e.g., Ho et al., 2002) and the observed unique associations with Chinese literacy in typical readers (e.g., Liu and Zhu, 2016), we added a measure of rapid naming to determine whether it would predict literacy skills in Chinese early adolescents and distinguish between typical readers and those with dyslexia.

Visual-orthographic knowledge is another construct we included in this study, as it is said to distinguish between children with and without dyslexia in various alphabetic and non-alphabetic orthographies (e.g., Wolf, 1999; Ho et al., 2002). Known to tap the processing of orthographic information that alters the unit of perception, visual-orthographic knowledge enables the reader to move from processing, e.g., particular letters to particular sequences of letters (Chung et al., 2010). In Chinese, visual-orthographic knowledge refers to the learners' sensitivity to conventional rules when structuring Chinese characters, including their competency in distinguishing a set of non-characters, visual symbols and pseudocharacters from real Chinese characters. Previous studies show that visual-orthographic knowledge uniquely predicts Chinese literacy skills in both typical readers (e.g., Tong et al., 2009) and those with dyslexia (e.g., Chung et al., 2011). Cognitive profiling studies also show that visual-orthographic knowledge tasks distinguish students with dyslexia from those without dyslexia during primary grades (e.g., Ho et al., 2002, 2004; Chung et al., 2010). Thus, we included this measure in our study.

We also included the measure verbal short-term memory (STM) because of its demonstrated importance for reading acquisition in both alphabetic and non-alphabetic languages (e.g., Savage and Frederickson, 2006; Kormos and Sáfár, 2008). Research in alphabetic scripts further indicates that poor readers tend to struggle when rehearsing, storing, encoding, and recovering speech stimuli from memory (e.g., Siegel and Ryan, 1988). Studies done in Chinese children and early adolescents with and without dyslexia (e.g., Ho et al., 2000; Chung et al., 2010) indicate that those with dyslexia performed worse on verbal STM measures of digit memory as well as other intricate memory tasks than did typically developing children. We included the verbal STM measure in the current study due to observed associations between this construct and Chinese literacy skills (e.g., Shu et al., 2006; Chung et al., 2011) and to ascertain its capacity to discriminate between good and poor readers in Hong Kong.

The last measure we included involves oral vocabulary. Some studies have suggested that because of the breadth of oral language deficits associated with dyslexia, the explanation offered by the phonological deficit hypothesis for reading failure is incomplete (e.g., Gallagher et al., 2000). Dyslexia is often viewed as a “general verbal limitation” that, over time, presents varying expressions and is on a continuum with multi-componential language deficits. Indeed, children identified as having dyslexia have exhibited an early history of rather inferior vocabulary skills, including other verbal deficits such as verbal comprehension (Scaborough, 1991; Gallagher et al., 2000). In Chinese, however, the role of oral language skills in reading development has not been extensively examined, except in a few studies where vocabulary knowledge predicted Chinese character recognition (e.g., Wang et al., 2006; Liu and McBride-Chang, 2010). Additionally, vocabulary knowledge distinguished readers with and without dyslexia and explained literacy skills in a study by Shu et al. (2006) in Mainland China. Thus, we included the oral vocabulary measure in our study to see whether it would be associated with Chinese literacy skills and to distinguish between good and poor readers in early adolescence.

### The present study

The research we have highlighted above shows that there are various cognitive-linguistic skills that distinguish readers with dyslexia in many languages. It is, however, important to investigate how deficits in morphological awareness in relation to the other five cognitive-linguistic skills are associated with reading difficulties as children with dyslexia graduate to senior primary grades and the extent to which such deficits persist. Questions then arise in view of the morphological nature of the Chinese language to determine the extent to which morphological awareness, among other cognitive-linguistic skills, is key in predicting Chinese word reading and dictation as readers with and without dyslexia transition into early adolescence. Also important is to establish the cognitive-linguistic deficits that persist into early adolescence for Chinese students with dyslexia.

The present study, therefore, sought to understand literacy development and impairment among Hong Kong Chinese early adolescent readers by investigating the potential importance of morphological awareness in explaining Chinese word reading and writing skills in readers with and without dyslexia from senior primary grades. We examined the extent to which six cognitive-linguistic skills—morphological awareness, phonological awareness, visual-orthographic knowledge, rapid naming, verbal STM, and oral vocabulary knowledge—would distinguish early adolescent readers with dyslexia from their peers with the same chronological age (CA). We also examined the relationships among the cognitive-linguistic skills, word reading and dictation and whether these relationships were the same for readers of dyslexia and typical readers.

## Methods

### Participants

One hundred and eight Hong Kong Chinese senior primary school students in Grades 5 and 6 were recruited for 2 groups, i.e., the group for adolescents with dyslexia and the CA control group. All participants were native Cantonese speakers and enrolled in local primary schools where the main medium of instruction was Cantonese. For literacy acquisition instruction, Hong Kong public schools do not provide phonics instruction. Thus, Cantonese reading instruction employs the “look and say” method, where learners typically memorize the characters (Holm and Dodd, 1996). The dyslexia group consisted of 54 students from Grade 5 to 6 [mean age = 130.70 months, standard deviation (*SD*) = 6.55], with 31 boys and 23 girls. The students who participated in this study had been assessed on Hong Kong standardized intelligence tests by qualified psychologists. These tests include the Hong Kong Test of Specific Learning Difficulties in Reading and Writing for Primary School Students [HKT-P(II)] (2nd ed.) (Ho et al., 2007) and the Hong Kong Wechsler Intelligence Scale for Children (HK-WISC) (Hong Kong Psychological Corporation, 1981). This test battery involved literacy tasks, phonological awareness, rapid naming, orthographic skills and phonological memory. The HKT-P(II) is a standardized test with local norms and is used to diagnose developmental dyslexia among students in Hong Kong primary schools. Participants with dyslexia had to meet the criterion that the literacy composite score, including one or more of the cognitive-linguistic composite scores in the HKT-P(II), was at least 1 standard deviation below their respective age. In line with the Hong Kong diagnostic criteria of developmental dyslexia, those with dyslexia also needed to have normal intelligence, with an IQ of 85 or above. Further screening of all participants was performed to ensure each had been accorded sufficient learning opportunities and without serious behavioral or emotional problems, suspected brain damage or uncorrected sensory impairment. Also excluded from our study were new immigrants.

In the control group, we recruited 54 typically developing readers from two Hong Kong primary schools. Based on age, 26 boys and 28 girls (mean age = 129.46 months, *SD* = 5.57) from the control group were matched to the dyslexic students (see Table 1). The control group members were all average performers, and based on the previous grade average, they were nominated to the group by their class teachers. The grade point average was within the 50–75 percentile for Chinese language/literature. None of the students had a childhood history of learning difficulty or psychopathology.

**Table 1:** Means, standard deviations, and *t*-tests of all variables for adolescent readers with dyslexia and typical readers.

	**Reliability**	**Dyslexics *N* = 54**		**Typical readers *N* = 54**		***t* tests**	**Effect size (*d*)**
		**Mean**	***SD***	**Mean**	***SD***	***t* values**	
Age in months		130.70	6.55	129.46	5.57	−1.06	−0.01
**LITERACY SKILLS**							
Chinese Word Reading	0.97	60.37	16.72	106.20	12.85	15.97^***^	0.53
Chinese Word Dictation	0.96	37.43	6.04	62.20	11.52	14.00^***^	0.48
**COGNITIVE-LINGUISTIC SKILLS**							
Rapid digit naming	0.93	39.08	7.33	26.91	4.99	−10.08^***^	−0.36
Verbal STM	0.79	59.87	12.46	72.43	13.38	5.05^***^	0.19
Morpheme discrimination	0.78	9.52	2.79	12.35	2.33	5.73^***^	0.26
Morpheme production	0.88	9.33	2.19	12.52	1.42	8.96^***^	0.29
Visual-orthographic knowledge	0.82	12.85	2.32	15.28	1.68	6.23^***^	0.17
Vocabulary knowledge	0.79	9.78	3.56	12.09	3.67	3.33^***^	0.21
Phonological awareness	0.87	13.48	3.04	14.00	2.69	0.94	0.04

*Test-retest reliability was computed for the rapid digit naming measure. Non-Dyslexics were matched on chronological age*.

*** *p < 0.001*.

### Materials and procedures

Participants were administered 9 measures, including the standardized vocabulary test from the HK-WISC, two morphological awareness tasks, two literacy tests, one test of visual-orthographic knowledge, one test of rapid naming, one test of phonological awareness, and a verbal STM task. The measures were all individually administered. Before formal testing, the participants were given two practice trials for each task. Written and informed consent from the parents/legal guardians of all participants was obtained prior to testing, and the Education University of Hong Kong's Human Research Ethics Committee approved the study. All measures were administered by trained experimenters.

### Assessment measures

#### Literacy

##### Chinese word reading

This measure was modeled on a test employed in previous studies (Chung et al., 2010, 2014). A total of 124 Chinese two-character words were selected as sample items from popular Chinese language textbooks recommended for use in upper primary school by the Hong Kong Education Bureau. These words were cross checked with the list of graphemes of commonly used Chinese characters (常用字字形表) that comprise 4762 commonly used words recommended by the Hong Kong Education Bureau (Curriculum Development Institute, 2007) for teaching at junior and senior primary school levels. These word items were presented in order of increasing difficulty, and students were asked to read the words aloud one-by-one. One point was awarded when the two characters in presented words were correctly read. The maximum score for this test was 124.

##### Chinese word dictation

This measure was modeled after tests from previous studies (e.g., Chung et al., 2010). Students were orally presented with 96 two-character words and then requested to write them down. Again, the target items were selected from three sets of Chinese language textbooks commonly used in upper primary schools. These words were also cross-checked with the list of graphemes of commonly-used Chinese characters (常用字字形表) used in primary school grades. For each correctly written character, one point was awarded. The maximum score for this test was 96.

#### Morphological awareness

##### Morpheme discrimination

The students' comprehension of a morpheme having different meanings in two morphemic words was measured using this task. This measure, which was constructed based on the test used in Chung et al. (2014) study, involved 19 items, each comprising four two-character words presented orally and visually. Each set had a character sharing the same word and written form but did not share the same meaning when joined with the other characters. For example, the character 信,/seon3/ was the common character in the words 信任/seon3 jam6/, trust; 信封, /seon3 fung1/, envelope; 信件, /seon3 gin6/, letter; and 信箱, /seon3 soeng1/, mailbox. For every presented set, participants were asked to identify the “odd” word. Thus, the correct answer was 信任, /seon3 jam6/, trust because the character /seon3/ in the word /seon3 jam6/ signified a dissimilar morpheme. One point was awarded for each correct answer, and 19 was the maximum score.

##### Morpheme production

This task, also used in the previous study (Chung et al., 2014), examined the participants' competence in applying and integrating the contextual and morphological information in particular settings. Participants were orally presented with 18 sentences with missing words. They were told to listen carefully as incomplete sentences would be read out to them and they would then be asked to fill the “blank” with a suitable word. An example sentence is “因為工作需要, 我在昨天購買了一台新的電____來處理文件”, *Due to my work, I brought a _______ home yesterday to process documents. One* of the possible correct responses for this was 電腦, *computer*, because 電, *electricity*, was joined to the word 腦, *brain*. When students provided a word response that satisfied the semantic constraints of the position and was sensible given the sentence context, a correct answer was awarded. The maximum score for the task was 18.

#### Rapid naming

Rapid Digit Naming. When presented with a paper having five stimulus items, i.e., digits (2, 4, 6, 7, 9) in different orders across five rows, the students were asked to name the numbers presented on the list as fast as they possibly could. Every participant performed this task twice, and the score was the average time to name the digits across the two trials.

#### Phonological awareness

A syllable and onset deletion task was used (McBride-Chang et al., 2003). In the syllable deletion task, the participants were orally presented with nine meaningless three-syllable items and were asked to say aloud what was left if one syllable was removed for each item. An example item was “say /fu1 on3 baan2/ without /baan2/,” and the correct answer was “/fu1 on3/.” In the onset deletion task, 10 real and four pseudo one-syllable words as well as four two-syllable and four three-syllable pseudo words (i.e., 22 items altogether) were used. Again, children were orally presented with each item and asked to remove the onset from each syllable. An example item was “say /tau1/ without /t/,” and the correct answer was “/au1/.” One point was allotted for each correct answer. A phonological awareness score was computed by summing the scores from the two tests.

### Visual-orthographic knowledge

#### Character matching

Constructed in line with Chung et al. (2010), this task assessed the learners' knowledge about the structures of Chinese characters. After looking at a target character, participants were asked to identify the same character from given options of nine stimuli comprising similar visual forms and orthographic units as the target characters. Made up of a combination of five error types, the stimuli involved inverted components, illegal positions, incorrect number of strokes, incorrect orientation and one component combining different components. For example, the target character 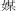 was simultaneously presented together with options such as 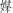 (one component merging with a dissimilar component), 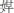 (one component with inappropriate strokes), 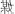 (the components in wrong positions), 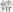 (a component with mirror orientation), and 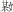 (components with reversed left/right positions). The maximum score was 18, and one point was awarded for correct answer.

### Verbal STM test

Non-word repetition. Adopted from previous studies (Chung et al., 2010, 2014), this measure tested the participants' phonological working memory. This memory test had 20 trials of three to eight Chinese syllables altogether. Although the particular presented syllables were legal phonetic syllables in Cantonese, they were also monosyllabic non-words (e.g., /bei5/, /tan5/, and /daai5/). A CD player was used to present stimuli, and the participants were asked to orally repeat the syllables, maintaining their order of presentation. A point was awarded for each correctly reproduced syllable.

### Vocabulary knowledge

#### Oral vocabulary

Adapted from Chik et al. (2012), this vocabulary test measured participants' ability to use vocabularies in a given context as well as the depth of their vocabulary knowledge. Participants were orally presented with nine Chinese two-character words (e.g., adjectives, verbs, and nouns) familiar to students in junior secondary school and were then requested to explain or define the given target words. Students were also asked to construct sentences using the target word to help illustrate what the word entails. For instance, given the target word (miracle), the likely words to define this would be (extraordinary), (unimaginable), (mysterious), and (very surprising and unexpected event). An example of a sentence would be (I was so sure my dog would never make it to the finish line in this event. Considering his ill health, just him coming this far is a pure miracle!). Three points were used to award a score: a point for clearly stating what the target word means, another for expounding on the meaning of the target word and one last point for using the target word in a suitable context. The maximum score was 18.

## Results

Three types of analyses are presented. First, the means, standard deviations (*SD*) and *t*-values of all tests are presented for both the group with dyslexia and the CA control group. Second, separate correlational associations for the two groups on their Chinese word reading and dictation are presented. Finally, associations of independent cognitive-linguistic measures with Chinese word reading and dictation for the two groups are explored using four-stage hierarchical regressions.

Coefficient alphas were calculated for each measure used in the present study. The reliability estimates for the rapid naming task involved test-retest reliabilities. The internal consistency reliabilities for all other tasks are presented in Table 1.

### Group comparisons of literacy and cognitive-linguistic measures

The means, standard deviations and effect sizes for all tasks are presented in Table 1. The reliability of the tasks was generally acceptable (all 0.70 and above). Independent *t*-tests were conducted to examine group differences on the measures of word reading and dictation. Although no significant mean difference was observed for the phonological awareness task, the observed mean differences for both literacy tasks and for morphological awareness, rapid naming, visual-orthographic knowledge, verbal STM, and vocabulary knowledge were all significant. The group with dyslexia performed worse than the CA across all these tasks except for the phonological awareness task [(*m* = 13.48, *sd* = 3.04), *t*_(106)_ = 94, *p* > 0.05], where no group differences were observed.

### Correlations between the literacy and cognitive-linguistic measures

As shown in Table 2, separate correlational analyses were conducted for early adolescent readers with and without dyslexia regarding their literacy and cognitive-linguistic skills. For both groups, after controlling for the effect of age, the early adolescent readers' Chinese word reading and dictation was significantly associated, that is, *r* = 0.27, *p* < 0.05 for the group with dyslexia and *r* = 0.33, *p* < 0.05 for the control group. In the two groups, the measures of morphological awareness were significantly associated with word reading and dictation skills. Further examination of the associations of the key cognitive-linguistic skills as regards Chinese word dictation showed different patterns in the two groups. For the group with dyslexia, the dictation task was significantly associated with visual-orthographic knowledge (*r* = 0.37, *p* < 0.01; see Table 2). However, for the control group, the Chinese dictation task was significantly associated with rapid naming (*r* = −0.38, *p* < 0.01).

**Table 2:** Correlations among all measures in readers with dyslexia (lower left) and typical readers (upper right) after statistically controlling for age in months.

**Measures**	**1**	**2**	**3**	**4**	**5**	**6**	**7**	**8**	**9**
1. Chinese word reading	–	0.33^*^	−0.22	0.17	0.39^**^	0.32^*^	0.15	0.05	−0.05
2. Chinese word dictation	0.27^*^	–	−0.38^**^	0.07	0.31^*^	0.32^*^	0.11	0.12	0.15
3. Rapid digit naming	−0.25	−0.13	–	0.07	−0.02	−0.11	0.06	−0.02	0.08
4. Verbal STM	0.20	0.04	−0.27^*^	–	0.11	0.19	0.08	0.05	0.25
5. Morpheme discrimination	0.36^**^	0.36^**^	0.09	−0.24	–	0.13	−0.01	0.02	0.06
6. Morpheme production	0.31^*^	0.33^*^	−0.22	0.16	−0.03	–	0.09	0.06	0.04
7. Visual-orthographic knowledge	0.26	0.37^**^	−0.25	−0.12	0.19	0.26	–	−0.15	0.02
8. Vocabulary knowledge	0.10	0.22	−0.02	−0.26	0.24	0.19	0.16	–	0.00
9. Phonological awareness	0.05	−0.08	−0.09	−0.05	0.19	−0.14	0.10	0.11	–

* p < 0.05;

** *p < 0.01*.

### Relations among cognitive-linguistic skills, word reading, and dictation

To examine the cognitive-linguistic skills that would explain Chinese word reading and dictation for early adolescent readers in the group with dyslexia and the typical readers, separate four-stage hierarchical regression analyses were conducted. Cognitive-linguistic tasks, including rapid naming, vocabulary knowledge, visual-orthographic knowledge, phonological awareness, and morphological awareness, were entered as independent variables at step 3 after controlling for age and the group interaction effect in steps 1 and 2, respectively. To differentiate group properties when explaining the associations between the variables in the regression models, dummy variables were created with the group with dyslexia as the reference group (typical readers = 0, dyslexic readers = 1). Thus, the interaction of the group and the cognitive-linguistic measure was entered at stage 4. Unfortunately, after this, the group interaction effect ceased to be significant, and the key skills known to explain Chinese literacy skills predicted neither of the assessed literacy measures in the group of adolescents with dyslexia (see Tables 3, 4). The results of step 3 of the regression analyses (refer to Tables 3, 4), therefore, indicated that after controlling for age and group interaction effects, only the constructs rapid naming and morphological awareness could significantly explain Chinese word reading and dictation in the two groups. The verbal STM, vocabulary, visual orthographic knowledge and phonological awareness measures were not associated with word reading and dictation.

**Table 3:** Hierarchical regression analyses explaining Chinese word reading.

	***R*^2^**	**Δ*R*^2^**	***F* Change**	**β**	***t***
Step 1	−0.01	0.00	0.11		
Age in months				−0.03	−0.33
Step 2	0.70	0.71	255.96^***^		
Age in months				0.05	1.03
Typical readers vs. Dyslexics				−0.85	−16.00^***^
Step 3	0.78	0.09	6.19^***^		
Age in months				0.03	0.58
Typical readers vs. Dyslexics				−0.45	−5.70^***^
Rapid digit naming				−0.14	−2.14^*^
Verbal STM				0.10	1.86
Morpheme discrimination				0.24	4.42^***^
Morpheme production				0.16	2.39^*^
Visual-orthographic knowledge				0.07	1.20
Vocabulary knowledge				0.01	0.10
Phonological awareness				−0.03	−0.70
Step 4	0.77	0.00	0.28		
Age in months				0.02	0.43
Typical readers vs. Dyslexics				−0.57	−0.80
Rapid digit naming				−0.10	−1.26
Verbal STM				0.06	0.82
Morpheme discrimination				0.21	2.50^*^
Morpheme production				0.15	1.28
Visual-orthographic knowledge				0.09	0.92
Vocabulary knowledge				0.02	0.30
Phonological awareness				−0.06	−0.76
Dummy_VSTM				0.17	0.65
Dummy_MP				0.03	0.10
Dummy_MD				0.11	0.55
Dummy_RDN				−0.14	−0.63
Dummy_V-OK				−0.14	−0.39
Dummy_PA				0.10	0.43
Dummy_VK				−0.06	−0.38

*VSTM, Verbal STM; MP, Morphological production; MD, Morphological discrimination; RDN, Rapid digit naming; V-OK, Visual-orthographic Knowledge; PA, Phonological Awareness; VK, Vocabulary Knowledge*.

* p < 0.05;

*** *p < 0.001*.

**Table 4:** Hierarchical regression analyses explaining Chinese word dictation.

	***R*^2^**	**Δ*R*^2^**	***F* Change**	**β**	***t***
Step 1	−0.01	0.00	0.00		
Age in months				0.00	0.00
Step 2	0.65	0.66	200.04^***^		
Age in months				0.08	1.45
Typical readers vs. Dyslexics				−0.81	−14.14^***^
Step 3	0.71	0.08	4.02^***^		
Age in months				0.06	1.06
Typical readers vs. Dyslexics				−0.44	−4.85^***^
Rapid digit naming				−0.16	−2.11^*^
Verbal STM				0.02	0.34
Morpheme discrimination				0.19	3.13^**^
Morpheme production				0.15	2.04^*^
Visual-orthographic knowledge				0.08	1.20
Vocabulary knowledge				0.04	0.74
Phonological awareness				0.02	0.27
Step 4	0.71	0.02	1.16		
Age in months				0.05	0.93
Typical readers vs. Dyslexics				0.79	1.00
Rapid digit naming				−0.19	−2.13^*^
Verbal STM				−0.04	−0.48
Morpheme discrimination				0.25	2.69^**^
Morpheme production				0.33	2.52^*^
Visual-orthographic knowledge				0.14	1.27
Vocabulary knowledge				0.08	1.09
Phonological awareness				0.12	1.46
Dummy_VSTM				0.18	0.63
Dummy_MP				−0.56	−1.68
Dummy_MD				−0.17	−0.78
Dummy_RDN				0.09	0.37
Dummy_V-OK				−0.13	−0.34
Dummy_PA				−0.45	−1.66
Dummy_VK				−0.05	−0.31

*VSTM, Verbal STM; MP, Morphological production; MD, Morphological discrimination; RDN, Rapid digit naming; V-OK, Visual-orthographic Knowledge; PA, Phonological Awareness; VK, Vocabulary Knowledge*.

* p < 0.05;

** p < 0.01;

*** *p < 0.001*.

Worth highlighting is that both measures of morphological awareness uniquely predicted Chinese word reading and dictation after controlling for age and group membership. As shown in Table 3, morpheme discrimination (β = 0.24, *p* < 0.001) and morpheme production (β = 0.61, *p* < 0.05) uniquely explained Chinese word reading. Similarly, morpheme discrimination (β = 0.19, *p* < 0.01) and morpheme production (β = 0.15, *p* < 0.05) uniquely predicted dictation skill. Overall, the model explaining Chinese word reading had a total variance of 78%, while that explaining dictation accounted for 71%.

## Discussion

The present study investigated the potential key role of morphological awareness in enhancing literacy development and impairment in early adolescent Chinese readers, as well as the extent to which the cognitive-linguistic profile of readers with dyslexia differed from typically developing readers in Hong Kong. We were also interested in establishing the link between cognitive-linguistic skills and literacy measures that may distinguish early adolescent readers with and without dyslexia. Our results provide data that add to the body of research evidence highlighting the potential importance of morphological awareness in literacy development and impairment (e.g., Carlisle, 1995), especially in early adolescent Chinese readers (Shu et al., 2006). Hierarchical regression analyses demonstrated that both morphological awareness tasks, including the rapid naming measure, uniquely predicted Chinese word reading and dictation even after controlling for age and group membership. In line with our second goal, we observed that the group with dyslexia was significantly weaker than the control group in the two literacy tasks, including the six cognitive-linguistic tasks: morphological awareness, visual-orthographic knowledge, rapid naming, phonological awareness, verbal STM, and vocabulary knowledge. Confirming existing work on the multiple cognitive-linguistic deficits (e.g., Shaywitz et al., 1999; Wolf and Katzir-Cohen, 2001) that may influence reading and dictation mechanisms in Chinese readers, our findings further indicate that even in early adolescence, there are still cognitive-linguistic and literacy differences between typical readers and those with dyslexia, indicating that certain cognitive deficits of dyslexia could persist through early adolescence (e.g., Shaywitz et al., 1999). Below, we highlight the potential importance of morphological awareness in literacy development and impairment among Chinese learners, and we shed light on the other cognitive-linguistic differences that distinguish readers with and without dyslexia in early adolescence.

### The role of morphological awareness

It is important to mention yet again that both of our measures of morphological awareness significantly differentiated between adolescent readers with and without dyslexia. The morphological awareness tasks were also significantly associated with Chinese word reading and dictation. There are a number of reasons why morphological processing in Chinese literacy development is important. First, as noted earlier, Chinese, by nature, has morphological characteristics (e.g., Packard, 2000) that can easily be associated with the practical aspects of reading impairment and development. Mastery in Chinese literacy is dependent on the salient semantic transparency of the compounding morphological structure in many words. This distinct compounding morphological structure coupled with the large number of Chinese homophones together suggest approaches and impairments that may tend to be more pronounced in Chinese readers.

Chinese has many homophones, in sharp contrast to the relatively few homophones in alphabetic languages. This makes phonological or sound information unreliable in identifying or decoding Chinese words. In fact, several studies have noted that this feature of morphological awareness is critical for Chinese word recognition at the word level (Shu et al., 2006; Tong et al., 2009). This is considering the fact that as readers become more competent, they tend to see more clearly how the morphemes are related to one another. By quickly grasping the homophonic nature of Chinese, that is, basically figuring out the fact that identical sounds may actually imply different meanings in varying word contexts, this realization is an advantage that not only enhances the competent reader's accuracy in Chinese word reading and dictation but also facilitates educated guesses (e.g., McBride-Chang et al., 2003; Shu et al., 2003). This is an important issue to highlight concerning Chinese literacy acquisition because, as would be expected, early readers search and desire to employ the obvious systematic and regular associations in their spoken and written language in order to facilitate proximal learning (Shu et al., 2006). For Chinese, this would clearly be the morphemic structure of the language.

Considering that the readers in our sample were from senior primary schools, this further highlights the key role of morphological awareness in literacy development across the primary grades. Bearing in mind also that our study was interested in identifying cognitive-linguistic skills that distinguish adolescent readers with dyslexia from the control group, it is possible that morphological awareness deficits impair reading development by slowing down the process of word reading, including dictation ability. This further highlights the likely difficulty Chinese readers with dyslexia may have in identifying and discriminating morphemes during literacy acquisition (e.g., McBride-Chang et al., 2003). Clearly, the immense quantity of homophones here presents a challenge that calls for extended hours of adult support and supervision (e.g., Li and Rao, 2000) compared with what would be necessary for acquiring literacy skills in an alphabetic orthography. This study shows that for early adolescent readers with dyslexia, attaining the acceptable levels of sensitivity to different homographs and the many homophonic morphemes for the purposes of skilled reading continues to be a great burden affecting both their reading and dictation skills (e.g., Shu et al., 2006; Chung et al., 2010). Even though literacy instruction in Hong Kong begins much earlier than in most other Chinese societies, difficulty in attaining levels of competence in reading could further be compounded by the fact that the traditional script the pre-readers are expected to learn is generally more complicated and contains much more visual information than the simplified script used in Mainland China (for details, see Wong et al., 2013; Kalindi et al., 2018). In general, the findings from this study indicate that the difficulty experienced by readers with dyslexia in morphological discrimination persists through the senior primary grades, as they still seem to have difficulty identifying and discriminating morphemes and in generalizing morpheme meaning. Together with findings from other studies (e.g., Shu et al., 2006; Liu and Zhu, 2016), the present findings indicate that persistent deficits in morphological awareness may largely affect the quality of semantic representations of morphemes, which, in turn, cause a vast number of homophonic and semantic errors in early adolescent readers with dyslexia. This is in line with what has been demonstrated in previous research (e.g., Xue et al., 2013; Liu and Zhu, 2016), that morphological awareness plays an important role in enhancing reading development not just for the early stages of acquiring reading skills (e.g., McBride-Chang et al., 2008) but more so across the grades.

Although our findings, in line with the study by Shu et al. (2006), highlight the critical role of morphological awareness with regard to literacy development and impairment among Chinese adolescents, it is worth noting differences such as the location of the study, methods of literacy instruction and the type of language and nature of the script to be learned (Cheung and Ng, 2003). These findings of the potential importance of morphological awareness in adolescence thus transcend script and literacy instruction method differences and call for further investigations into the likely importance of morphological awareness in other Chinese societies. The analyses of the current study demonstrated that morphological awareness uniquely explained literacy performance, and worth highlighting is that the two measures of morphological awareness both predicted Chinese word reading and dictation. This information might be useful in confirming the core cognitive-linguistic skills tapped into separately by the two morphological awareness tasks, i.e., morpheme discrimination and morpheme production.

### Other cognitive-linguistic skills differentiating between early adolescent readers with dyslexia and typical readers

We also observed some differences between those with dyslexia and the control group in other cognitive-linguistic skills. Besides the constructs of morphological awareness, our construct of rapid naming was strongly associated with both literacy tasks. Additionally, in line with previous studies (e.g., Chung et al., 2010, 2011), we observed that most adolescent readers with dyslexia were slower than the control group in rapid naming. The relatively arbitrary associations between print and sound in the Chinese script play a crucial role in Chinese reading development and impairment, thus making rapid naming important (e.g., McBride-Chang and Ho, 2000). Studies performed on Chinese children with dyslexia (e.g., Ho et al., 2002, 2004) indicate that rapid naming deficits are the most prominent of difficulties faced by Chinese readers with dyslexia. Consistent with the current findings, Chung and Ho (2010), Chung et al. (2010) found that rapid naming deficits continued to be among the dominant cognitive-linguistic deficits and a major difficulty experienced by adolescent Chinese readers with dyslexia. Additionally, the fact that rapid naming was uniquely associated with both word reading and dictation, even after controlling group membership and age, is noteworthy. This finding underscores, yet again, the importance of rapid naming in understanding the development of a wide range of reading-related skills in Chinese readers with and without dyslexia (Shu et al., 2006; Tong et al., 2009; Chung et al., 2011).

There were also substantial differences in the visual-orthographic knowledge of students with dyslexia and the control group. This construct of orthographic processing was moderately associated with Chinese character dictation in the group of adolescents with dyslexia. It is possible that adolescents with dyslexia could still be having difficulty with mastering knowledge of the Chinese orthographic structure as well as the radical positions. Indeed, considering that even typically developing children in Hong Kong have considerable challenges in acquiring comprehensive visual-orthographic knowledge of Chinese (e.g., Ho et al., 2004), it is likely that the problem could be even more pronounced for those with dyslexia. The generally complex traditional Chinese script used in Hong Kong likely compounds the visual-orthographic deficit problem among students with dyslexia. In line with previous research, the current data demonstrate that the visual-orthographic processing ability of early adolescents with dyslexia does not improve over time (e.g., Bruck, 1998). While a number of studies have also observed associations between visual-orthographic knowledge and literacy skills (e.g., Tong et al., 2009; Chung et al., 2011), the failure of visual-orthographic knowledge to predict literacy tasks has equally been reported in isolated cases (e.g., Liu and Zhu, 2016). In any case, the evidence of association calls attention to the importance of visual-orthographic knowledge in Chinese literacy development.

Although the constructs of vocabulary knowledge and verbal STM both significantly distinguished between the early Chinese adolescent readers with and without dyslexia, these constructs were not associated with the literacy measures for the control group or the group with dyslexia. The failure by the Verbal STM and vocabulary knowledge measures to be associated with literacy tasks is rather unexpected, considering the established importance of verbal STM (e.g., Zhang et al., 1998; Gathercole et al., 2004) and vocabulary knowledge (e.g., Shu et al., 2006) among Chinese readers. Considering that the observed effect sizes were relatively small and considering the lack of association between these constructs and the literacy measures, future studies should preclude this unexpected finding, perhaps by using a large sample size and including other measures, such as a backward digit span, for the verbal STM.

As for the measure of phonological awareness, it is unsurprising that it could neither differentiate between the two groups nor be associated with the literacy tasks (e.g., Ho et al., 2004; Chung et al., 2010) in the control group and the group with dyslexia. This is partly because a phonological awareness deficit has less frequently been reported compared to other cognitive-linguistic deficits, particularly for Hong Kong students with dyslexia. Also, considering the way that Hong Kong readers learn to read the characters using the whole word approach as opposed to the phonetic coding system used in Mainland China (reflecting the importance of phonological awareness, e.g., Shu et al., 2006), it is likely that phonemic awareness skills may not be well developed even for readers in upper primary school (Chung et al., 2010). Despite this finding among Hong Kong learners, previous studies have indicated the likely importance of phonological awareness in Chinese reading development among early learners (e.g., Ho and Bryant, 1997; McBride-Chang and Ho, 2000). Thus, more studies need to be conducted in order to shed more light on the nature of the association between phonological awareness and literacy skills in Chinese as well as the role of phonological awareness in the reading acquisition process of adolescent readers with and without dyslexia.

### Limitations and future directions

There are a number of limitations to the present study. First, the tasks presented in this study only measured accuracy and did not limit the response time for the tasks. Since readers with dyslexia generally show poorer performance on tasks measuring speed of processing, it is likely data on their reaction time would have helped reveal cognitive-linguistic processing at the lexical level. Second, the data were limited to readers from Hong Kong. Considering the differences obtaining in various Chinese societies concerning the nature of the script used, i.e., traditional vs. simplified, as well as the different modes of literacy instruction adopted with Chinese learners in Hong Kong, Mainland China, and Taiwan, the extent to which our results are generalizable across Chinese societies remains unclear. Third, the present study showed that verbal STM, vocabulary knowledge, and phonological awareness appeared to be a less important feature in Hong Kong adolescent readers with dyslexia. This calls for further investigation where additional measures of a particular construct could be added, especially for verbal STM, as this would enhance understanding of the impairments of verbal working memory in relation to language skills. As regards phonological awareness, a further examination of the nature of phonological problems experienced by Chinese readers using a phonemic coding system may be worth exploring. Fourth, the current study had limited and specific sets of cognitive-linguistic skills that may not be comprehensive enough to enhance understanding of reading and writing problems in Chinese. Other cognitive-linguistic skills, such as syntactic skills, could be examined in future studies. Fifth, only students in relatively higher grades were included as participants, with all measures administered at a single time point. Inasmuch as our study established strong associations between morphological awareness and literacy skills, no causal links can be determined, hence the need for future studies to turn to the causal nature of this association through longitudinal studies.

Despite these limitations, the present study is valuable firstly because the findings are linked to the aims of this special issue. More importantly, it is among the few studies that have considered the potentially unique role of morphological awareness, among other cognitive-linguistic correlates, in distinguishing early adolescent Chinese readers with and without dyslexia. For Chinese early adolescent readers, with or without dyslexia, it appears that there is a somewhat natural reliance on morphological awareness for the acquisition of Chinese literacy skills. In addition to studies on younger (e.g., McBride-Chang et al., 2003) and older (e.g., Shu and Anderson, 1997) developing Chinese readers, the current findings demonstrate the need to further explore the concept of morphological awareness in understanding reading development and impairment for Chinese adolescent readers. Although studies of cognitive profiles distinguishing between Chinese readers with and without dyslexia have mostly been performed with children (e.g., Ho et al., 2002, 2004), the present study has revealed that readers with dyslexia in Chinese continue to display problems in reading and writing even when in upper primary school, indicating that dyslexia is a difficulty that persists chronically across all ages and scripts.

## Ethics statement

This study was carried out in accordance with the recommendations of Education Guidelines on Ethics Research, by the University's Institutional Review Board i.e., Human Research Ethics Committee of the Education University of Hong Kong, with written informed consent from all subjects. All subjects gave written informed consent in accordance with the Declaration of Helsinki. The protocol was approved by the Human Research Ethics Committee.

## Author contributions

KC and SK both made substantial contributions to the design of the work, acquisition of the data, analysis, as well as the write-up of the manuscript.

### Conflict of interest statement

The authors declare that the research was conducted in the absence of any commercial or financial relationships that could be construed as a potential conflict of interest.
